# Clinical Study on the treatment of chronic infectious ulcers using Platelet-Rich technology combined with moist dressings

**DOI:** 10.12669/pjms.40.7.8468

**Published:** 2024-08

**Authors:** Xu Zhang, Zhigang Lang

**Affiliations:** 1 Xu Zhang, Department of Orthopedics, Sichuan Provincial Orthopedics Hospital, Chengdu 610000, Sichuan, China; 2 Zhigang Lang, Department of Orthopedics, Sichuan Provincial Orthopedics Hospital, Chengdu 610000, Sichuan, China

**Keywords:** Platelet-rich Plasma, Moist Dressings, Chronic Infectious Ulcers, Wound Healing

## Abstract

**Objective:**

To explore the clinical efficacy of platelet-rich technology combined with moist dressings in the treatment of chronic infectious ulcers.

**Methods:**

This was a retrospective study. The subjects of the study were 48 patients with chronic infectious ulcers in Sichuan Provincial Orthopedics Hospital from January 2019 to June 2022. Enrolled patients were randomly divided into four groups(n=12), and received different treatment methods respectively. Further analysis and comparison were performed on the changes in wound volume, wound healing status, wound bacterial culture results, and the incidence of adverse reactions among the four groups.

**Results:**

Three months after debridement, the wound volume of all four groups of patients was significantly reduced compared with that before debridement, with a statistically significant difference in intra-Group-Comparison(*P*<0.05). The inter-Group-Comparison revealed a statistically significant difference in wound volume in Group-A, Group-B, and Group-C than that in Group-D(P<0.05). After treatment, the wound healing status of patients in groups A, B, and C was significantly better than that of patients in Group-D, with a statistically significant difference(*P*<0.05). During treatment, patients in all four groups had decreased count of would bacteria, and showed negative results of wound bacterial culture by the three-month follow-up. No serious adverse reactions were observed in the four groups during treatment, and all improved after management, with no statistically significant difference in the incidence of adverse reactions(*P*>0.05).

**Conclusion:**

Platelet-rich technology combined with moist dressings may effectively promote the repair of chronic infectious ulcer wounds, with good clinical safety.

## INTRODUCTION

Chronic ulcers refer to ulcers that take more than one month to heal. This disease often involves infection, and is a common and frequently occurring condition in surgery, mainly affecting middle-aged and elderly people. Its progression is typically slow and challenging to treat, with high costs and a tendency for recurrence, and may even cause local malignancy in severe cases. Patients not only suffer from the torment of the disease and the heavy economic burden but also experience a significant impact on their work and life.^1^,^2^ Chronic ulcers have a complicated etiology, and are generally attributed to diabetes, trauma, burns, local tissue compression, and ischemic hypoxia.^3^

The treatment of chronic infectious ulcers remains a challenging issue in clinical practice, with treatment methods mainly including active control of the primary disease, debridement and skin grafting, regular dressing changes, vacuum sealing drainage(VSD), surgical flap repair, anti-infection treatment, etc.^4^ However, these therapeutic approaches have several disadvantages, such as prolonged treatment cycles, considerable expenses, and a failure to address fundamental issues such as tissue necrosis and ulceration.^5^ The concept of autologous platelet-rich plasma(PRP) was proposed for the first time by Whitman in 1997.^6^ Since then, it undergoes continuous improvement in its preparation, with gradual expansion in its range of applications. Owing to the release of growth factors, chemokines, and cytokines by activated platelets, PRP can promote collagen synthesis, fibrous tissue and granulation tissue survival, and stimulate wound tissue regeneration and repair.

Currently, PRP has been applied extensively in fields such as orthopedics, oral and maxillofacial surgery, and burn surgery, with the achievement of favorable clinical outcomes. It may be considered to be a new alternative for the treatment of chronic infectious ulcers. Multiple clinical studies have confirmed the significant efficacy of PRP in treating chronic non-healing wounds and diabetic foot ulcers.^7^,^8^ However, there are few studies on PRP in the treatment of chronic infectious ulcers. In view of the above, the present clinical study was performed to observe the effect of PRP for treating chronic infectious ulcers, with the purpose to explore and evaluate the effectiveness of PRP in treating this disease, so as to provide evidence for the clinical application of PRP in chronic infectious ulcers.

## METHODS

This was a retrospective study. The subjects of this study were 48 patients with chronic infectious ulcers treated in Sichuan Provincial Orthopedics Hospital from January 2019 to June 2022. Data were retrieved from the hospital information and management system. Collect their various information of all patients. Included patients were randomly divided into four groups, with 12 cases in each group.

### Ethical Approval:

The study was approved by the Institutional Ethics Committee of West China Hospital, Sichuan University (No.: 2017-6-30-1; date: June 30, 2017), and written informed consent was obtained in retrospect after discharging the patient.

### Inclusion criteria:

- Patients who met the criteria for chronic infectious ulcers.
- Patients with good tolerance to the drugs used in this study and high treatment compliance.
- Patients whose family members agreed and signed an informed consent form.

### Exclusion criteria:

- Patients with platelet dysfunction syndrome, severe thrombocytopenia, hemodynamic instability, or sepsis.
- Patients who had used non-steroidal anti-inflammatory drugs within 48 hours.
- Patients who had received local corticosteroid injections within one month.
- Patients who had discontinued systemic corticosteroid therapy for less than two weeks.
- Patients with malignant tumors, especially those with hematopoietic or skeletal system malignancies.
- Patients with hemoglobin levels <100 g/L and platelet counts <100 x 10^9^/L.
- Patients with coronary heart disease, diabetes and other basic diseases.

### PRP Preparation:

Under strict aseptic conditions, 31 mL of venous blood was collected from each patient in the morning, 1 mL of which was used for platelet counting, and the remaining 30 mL for PRP preparation. Anitua method (single centrifugation): Blood samples were subjected to centrifugation at 160 x g for six minutes, resulting in three layers in the tube, i.e., the supernatant in the upper layer, red blood cells in the lower layer is red blood cells, and a thin, pale-yellow layer at the interface of the two layers, which was the PRP layer. Landesberg method (two-step centrifugation): After the first centrifugation at 200 x g for 10 minutes, all the supernatant was collected to 3 mm below the interface, followed by a second centrifugation at 200 x g for 10 minutes, with the upper layer being platelet-poor plasma, and the remaining being the PRP layer after discarding approximately 3/4 of the supernatant. Aghaloo method (two-step centrifugation): PRP was obtained after the first centrifugation at 215 x g for 10 minutes, and the second centrifugation at 863 x g for 10 minutes. A certain amount of the prepared PRP (0.1 mL ) was drawn by a sterile syringe for platelet counting.

### Treatment Methods:

All four groups of patients received basic treatment(blood glucose and blood pressure control, neurotrophic treatment, circulation improvement, etc.) and routine treatment (timely wound debridement, decompression, drainage, and dressing changes). In Group-A, PRP with a platelet concentration enrichment factor of approximately 2-3, obtained using the Anitua method, was sprayed onto the wound surface combined with an activator; after coagulation, the wound was covered with Vaseline gauze and wrapped with gauze and a bandage. In Group-B, PRP with an enrichment factor of 5-6, obtained using the Landesberg method, was sprayed onto the wound surface combined with the activator, after which the wound was covered with Vaseline gauze, and wrapped with gauze and a bandage after coagulation. In Group-C, PRP with an enrichment factor of 7-8, obtained using the Aghaloo method, was sprayed onto the wound surface combined with the activator; after coagulation, the wound was covered with Vaseline gauze, and wrapped with gauze and a bandage. Group-D served as the blank control Group-and patients in this group only received moist dressing for wound coverage and dressing changes. PRP was applied once every two weeks in Groups A, B, and C, with a total of four treatments.

### Outcome measures:

***Wound volume changes:*** The wound volume was measured at baseline and at the three months follow-up using a sterile saline injection method. By using a 20 mL syringe, sterile saline was injected into the wound and filled from the deepest site to the wound edge. The volume of saline in milliliters was calculated and recorded as the wound volume. Wound coverage rate =(pre-treatment wound volume - post-treatment wound volume) / pre-treatment wound volume × 100%.

***Wound healing status:*** Wound healing was evaluated by the attending physician based on clinical experience and knowledge (poor, effective, significantly effective, and healed).

***Comparison of wound bacterial culture results:*** Bacterial cultures of wound secretions were performed before wound debridement in all patients. After three months of follow-up, another secretion culture was performed for cases with unhealed wounds for further comparison.

***Adverse reactions:*** The occurrence of drug-related adverse reactions, such as nausea, vomiting, diarrhea, rash, as well as liver and kidney dysfunction, was observed during the treatment period.

### Statistical Analysis:

Data were analyzed using SPSS 26.0 software. Measurement data with normal distribution were expressed by mean ± standard deviation(±*s*), and inter-Group-Comparisons were performed using one-way analysis of variance(ANOVA). The confidence interval was 95%. Counting data were represented by n(%), and comparisons between groups employed the chi-square () test. *P*< 0.05 was considered statistically significant.

## RESULTS

There were no significant differences in the baseline data of the patients with chronic infectious ulcers in the four groups (*P* > 0.05, Table-I), indicating comparability among groups. As shown in Table-II, there were no significant differences in wound volumes of the four groups before treatment (*P* > 0.05). After three months of wound debridement, wound volumes in all four groups were significantly reduced compared to those before debridement, with statistically significant intra-Group-Differences (*P* < 0.05). The inter-Group-Comparison revealed statistically significant differences in wound volumes in Groups A, B, and C than those in Group-D (*P* < 0.05).

**Table-I:** Comparison of general data among the four groups of patients.

Groups	Age (years, *±s*)	Gender (M/F, n)	Wound formation time (months, *±s*)	Wound volume before treatment (cm³,*®*±*S*)
Group-A (n=12)	62.83±2.04	9/3	16.17±0.94	3.09±0.37
Group-B (n=12)	62.42±1.56	8/4	15.75±0.75	2.75±0.27
Group-C (n=12)	63.83±1.75	8/4	16.08±0.90	2.81±0.64
Group-D (n=12)	64.67±2.84	7/6	15.67±0.65	3.07±0.38
*F/χ^2^* value	2.776	1.271	1.077	1.938
*p* value	0.052	0.736	0.369	0.137

**Table-II:** Comparison of wound volume changes among the four groups of patients.

Groups	Wound volume before treatment (cm^3^,*±s*)	Wound volume after treatment (cm^3^,±*S*)
Group-A (n=12)	3.09±0.37	1.88±0.50
Group-B (n=12)	2.75±0.27	1.76±0.49
Group-C (n=12)	2.81±0.64	1.73±0.58
Group-D (n=12)	3.07±0.38	2.27±0.29
*F* value	1.938	3.187
*p* value	0.137	0.033

After treatment, the wound conditions in Groups A, B, and C were significantly better than those in Group-D, with statistically significant differences (*P* < 0.05, Table-III). No obvious large-scale necrosis was observed in the wounds of the four groups after debridement and other treatments. At the end of the first treatment cycle, the wounds in Groups A, B, and C showed no obvious exudation, and the granulation tissue was red and fresh, with a granular appearance. In Group-D, there was still a small amount of inflammatory exudation, and the granulation tissue was red. At the three months follow-up, the wounds in Groups A, B, and C had no exudation, and the granulation tissue was abundant, red, full, and granular in appearance, while Group-D showed no exudation and less pink granulation tissue compared to Groups A, B, and C. At admission, all patients in the four groups had positive wound cultures. During treatment, patients in all four groups showed decreased bacterial counts of the wound surface, and by the three-month follow-up, all patients’ wound cultures were negative.

**Table-III:** Comparison of wound healing outcomes among the 4 groups of patients after treatment [cases (%)].

Groups	Poor	Effective	Significantly effective	Healed	Total effectiveness rate
Group-A (n=12)	0(0.00)	4(33.33)	8(66.67)	0(0.00)	12(100.00)
Group-B (n=12)	2(16.67)	1(37.50)	9(75.00)	0(0.00)	10(83.33)
Group-C (n=12)	2(16.67)	2(16.67)	8(66.67)	0(0.00)	10(83.33)
Group-D (n=12)	4(33.33)	6(50.00)	2(16.67)	0(0.00)	8(66.67)
*χ*^2^ value					13.094
*p* value					0.042

No severe adverse reactions were observed in the four groups during treatment, and all improved after treatment. There was no statistically significant difference in the incidence of adverse reactions among groups (*P* > 0.05, Table-IV).

**Table-IV:** Comparison of adverse reactions among the four groups of patients [cases (%)].

Groups	Allergy	Itching	Pain	Burning sensation	Infection exacerbation
Group-A (n=12)	0(0.00)	1(8.33)	0(0.00)	1(8.33)	0(0.00)
Group-B (n=12)	1(8.33)	1(8.33)	0(0.00)	0(0.00)	0(0.00)
Group-C (n=12)	1(8.33)	1(8.33)	0(0.00)	1(8.33)	0(0.00)
Group-D (n=12)	0(0.00)	1(8.33)	1(8.33)	1(8.33)	1(8.33)
*χ*^2^ value					1.297
*p* value					0.730

## DISCUSSION

Chronic infectious ulcers are difficult-to-healing chronic wounds, with slow healing and prolonged cycles of treatment.^9^ Therefore, outcome measures were analyzed in this study three months after treatment. According to the results, patients in the three groups applying PRP showed significantly higher wound healing rates than those in the control group, which is consistent with many research findings both domestically and internationally. It supports good nourishing and repairing effects of PRP on wounds. The white blood cells it contains can inhibit or kill pathogens, and remove necrotic tissues, thereby effectively exerting anti-infective effects.

Moreover, the fibrin in PRP can enhance the scaffold function for repairing cells, stimulating local tissue regeneration and reducing wound contraction. It has been reported^10^ that PRP can improve the quality of life of patients with chronic ulcers, and reduce wound treatment costs, thus enhancing patient treatment confidence, and reducing the occurrence of adverse reactions. In this study, adverse reactions occurred in all groups during treatment, which, however, were alleviated after treatment and no serious adverse events occurred. There was no significant difference in the incidence of adverse reactions among groups(*P*> 0.05). However, owing to a limited sample size in the present study, further statistical analysis based on expanded sample size is needed to determinates the occurrence of adverse reactions.

As a common and frequently-occurring disease in Surgery, the chronic infectious ulcer is characterized by a prolonged treatment cycle, difficult to cure, and a high risk of recurrence. Chronic infectious ulcers may produce great physical and psychological consequences to patients and their families, impairing patients’ ability to carry out daily activities and negatively affecting their quality of life.^11^ Common etiologies of chronic infectious ulcers include trauma, venous stasis ulcers, diabetic ulcers, bedsore, and radiation injuries.^12^ The main pathological factors involve changes in the wound microenvironment due to various factors, such as local tissue nutrient deficiency, decreased quality of growth factors, infiltration of inflammatory cells, impaired function of repair cells, and the presence of senescent cells, resulting in the protracted course of chronic infectious ulcers.^13^

In terms of its principal treatment mechanisms, it is possible to reduce vascular permeability at the ulcer site, improve local blood microcirculation, and promote the formation of new blood vessels, thereby increasing local concentration of growth factors at the ulcer site, and accelerating wound healing.^14^ However, conventional treatment for chronic infectious ulcers is time-consuming and expensive. PRP is obtained to be plasma with a high concentration of platelets, which can produce a vast array of growth factors and cytokines to increase the concentration of local growth factors at the ulcer site. This, in turn, promotes cell division, accelerates cell proliferation, and boosts local nerve repair and neovascularization of the ulcer site, thus initiating the ulcer healing process.^15^ In addition, PRP contains high concentrations of platelets and white blood cells, which is essential for its antibacterial effects.^16^ Granulocytes, monocytes and lymphocytes contained in white blood cells can exert antibacterial effects, while platelets release antibacterial substances, such as antimicrobial peptides, chemotactic factors, and peroxides. These substances can directly inhibit or kill pathogens.

At the same time, PRP can regulate the balance of matrix metalloproteinases and their inhibitors and can promote the restoration of senescent cells. Previous studies have shown^17^,^18^ that, compared with antibiotic treatment, PRP did not induce antibiotic resistance and had a synergistic effect in preventing infection. Moreover, PRP can effectively promote wound repair in patients with refractory diabetic foot ulcers without causing severe adverse reactions.^19^

### Limitations of the study:

However, no consensus has been reached concerning the methods for PRP preparation, and there are varied concentrations of PRP obtained using different methods. It remains unclear whether there are differences in the wound repair effect of PRP at different concentrations. Additionally, further research is also required to clarify the regulatory mechanisms of growth factor release in PRP and the regulatory mechanisms of growth factors on wound healing. Besides, some observation indicators were determined by the attending physicians based on their knowledge and clinical experience, which introduces a certain degree of subjectivity. Moreover, this study employed a sterile saline injection method to calculate wound volume, with the possibility of producing errors in actual operation. Collectively, findings in the present research should be confirmed based on large-scale and long-term profound study in the future.

## CONCLUSIONS

PRP is rich in platelets and white blood cells, which possesses certain anti-inflammatory properties, and may effectively promote the healing of chronic infectious ulcer wounds with minimal adverse reactions.

### Authors’ Contributions:

**LZ:** Designed this study and prepared this manuscript, and are responsible and accountable for the accuracy or integrity of the work.

**XZ:** Collected and analyzed clinical data.
